# Understanding the early cold response mechanism in IR64 *indica* rice variety through comparative transcriptome analysis

**DOI:** 10.1186/s12864-020-06841-2

**Published:** 2020-06-24

**Authors:** Pratiti Dasgupta, Abhishek Das, Sambit Datta, Ishani Banerjee, Sucheta Tripathy, Shubho Chaudhuri

**Affiliations:** 1 Division of Plant Biology, Bose Institute, P1/12 CIT Scheme VII M, Kolkata, 700054 India; 2 Structural Biology & Bioinformatics Division, CSIR- Indian Institute of Chemical Biology, Kolkata, 700032 India

**Keywords:** IR64, Cold shock, Transcriptome, Differentially expressed genes (DEGs), Transcription factors (TFs), Calcium signaling, Kinases, Redox signaling, DRE motif

## Abstract

**Background:**

Cellular reprogramming in response to environmental stress involves alteration of gene expression, changes in the protein and metabolite profile for ensuring better stress management in plants. Similar to other plant species originating in tropical and sub-tropical areas, *indica* rice is highly sensitive to low temperature that adversely affects its growth and grain productivity. Substantial work has been done to understand cold induced changes in gene expression in rice plants. However, adequate information is not available for early gene expression, especially in *indica* variety. Therefore, a transcriptome profile was generated for cold shock treated seedlings of IR64 variety to identify early responsive genes.

**Results:**

The functional annotation of early DEGs shows enrichment of genes involved in altered membrane rigidity and electrolytic leakage, the onset of calcium signaling, ROS generation and activation of stress responsive transcription factors in IR64. Gene regulatory network suggests that cold shock induced Ca2+ signaling activates DREB/CBF pathway and other groups of transcription factors such as MYB, NAC and ZFP; for activating various cold-responsive genes. The analysis also indicates that cold induced signaling proteins like RLKs, RLCKs, CDPKs and MAPKK and ROS signaling proteins. Further, several late-embryogenesis-abundant (LEA), dehydrins and low temperature-induced-genes were upregulated under early cold shock condition, indicating the onset of water-deficit conditions. Expression profiling in different high yielding cultivars shows high expression of cold-responsive genes in Heera and CB1 *indica* varieties. These varieties show low levels of cold induced ROS production, electrolytic leakage and high germination rate post-cold stress, compared to IR36 and IR64. Collectively, these results suggest that these varieties may have improved adaptability to cold stress.

**Conclusions:**

The results of this study provide insights about early responsive events in *Oryza sativa l.ssp. indica* cv IR64 in response to cold stress. Our data shows the onset of cold response is associated with upregulation of stress responsive TFs, hydrophilic proteins and signaling molecules, whereas, the genes coding for cellular biosynthetic enzymes, cell cycle control and growth-related TFs are downregulated. This study reports that the generation of ROS is integral to the early response to trigger the ROS mediated signaling events during later stages.

## Background

*Oryza sativa* L. ssp. *Indica* being a tropical crop is highly sensitive to low-temperature stress leading to impaired growth and massive losses in grain productivity. Reports suggest that rice plants are more susceptible to cold stress during the seedling, tillering, panicle development and flowering stages. Rice grown below the ambient temperature leads to a lower rate of germination, retarded seedling emergence, delayed vegetative growth, reduced rates of photosynthesis. Continued exposure to cold stress causes tissue necrosis and ultimately, cellular death [1–4]. Thus, the agronomic productivity of rice plants is heavily affected under low-temperature conditions, especially in the elevated regions where cold mountain water is used for irrigation.

After perceiving the cold stress, cells undergo increase in their membrane rigidity due to a reduction in the plasma membrane fluidity [5]. This rise in the membrane rigidity causes increased electrolytic leakage from the cell, which acts as the primary signal for triggering cold response via activation of the cold-responsive gene expression [6–9]. Studies have shown that increase in membrane rigidity activates early cytoplasmic signals, such as triggering the MAPK signaling cascade, and the influx of cytoplasmic Ca^2+^ via mechano-sensitive Ca^2+^ channel or ligand-gated Ca^2+^ channel [10]. The increase of cytoplasmic Ca^2+^ activates a myriad of downstream signaling pathways, mainly via calcium decoders such as calcium-dependent protein kinases (CDPKs), to further activate the transcription of cold-responsive transcription factor belonging to C-repeat binding factor (CBF)/ Dehydration responsive element binding (DREB) family [11]. The DREB transcription factor binds to DRE site in the promoter region, thereby activating the expression of many cold-responsive (COR) genes (such as LT1, KIN, RAB, ERD genes). These DREB regulon genes play an important role in stabilizing membrane structure, activating ROS scavengers, and promoting the production of osmoprotectants to protect both the membrane and organelle damage during cold stress [12–16]. Microarray analysis has identified other transcription factors such as HSF1C, ZAT12, ZF, ZAT10 and SZF2 that are co-expressed with CBF and can positively regulate COR gene expression to impart cold stress tolerance [17]. Other transcription factors have been reported in *Arabidopsis* such as Eskimo1 and HOS9, which also participate in freezing tolerance and together constitute the CBF independent regulation [18, 19].

High-throughput RNA sequencing data have contributed significantly to understanding the molecular mechanism of cold response in rice. Owing to the diverse growing conditions and availability of various rice cultivable varieties worldwide; it is integral to continue with the high-throughput study of different varieties. Such studies provide a better understanding of the complexity of cold signaling that greatly enhances growth and grain productivity. Moreover, the response to cold stress in rice varies with tissue type, as well as varying developmental stages. Further, more pronounced effects are observed during the seedling stage and flower development in rice plants, when subjected to cold stress conditions. Among the two major subspecies of rice, the *japonica* varieties, usually grown at higher altitudes, are more tolerant to cold stress compared to *indica* varieties which are typically grown in tropical regions [20–22]. Previous studies have shown that cold-responsive genes can be clustered under two major groups, i.e., regulatory protein-coding genes that perceive the signal and functional protein-coding genes which initiate the abiotic stress response [23, 24]. The genes coding for regulatory proteins includes signaling molecules, such as kinases, phosphatases, calcium-binding messenger molecules, transcription factors, micro RNAs, and Two-Component Systems [25–27]. The second cluster of functional protein-coding genes comprise antioxidants, players of ROS removal, compatible solutes, and other hydrophilic molecules crucial for maintaining the osmotic balance [27–30]. However, the expression of this group of genes majorly depends on the stress exposure time and tissue type.

Previous work has identified several cold-tolerant wild rice varieties, such as Dongxiang common wild rice, Chaling wild rice, and Guangxi wild rice [31–33]. These varieties have been reported to withstand temperatures as low as − 9 °C to − 12 °C. Shen et al., 2014, suggested that Dongxiang common wild rice is an ideal germplasm source for the generation of cold-resistance breeding [34, 35]. In the modern era, various approaches like hybridizations among wild rice varieties and novel genetic manipulation methods have been used to improve the crop’s growth and productivity under cold conditions. Cold tolerant introgressed lines, generated from *indica* variety, with *japonica* rice have shown increased tolerance to cold stress compared to cold-sensitive *indica* varieties [36]. Although strong selection pressure may be attributed to the evolution of such cold-tolerant genotypes, the molecular basis of tolerance in such intrinsically tolerant rice varieties is still a less explored field. In the present scenario, the knowledge of cold-responsive genes, and the cold-tolerant QTLs triggered at various stages, during cold stress can be exploited for breeding cold-tolerant rice varieties. *Indica* rice varieties with better adaptability to low-temperature conditions need to be identified. Furthermore, improving high yielding tropical *indica* varieties, such that they are better suited for lower temperatures, can provide a solution to the loss of yield in the paddy fields of high-altitude terrain. This study was aimed at identifying genes that undergo differential expression in response to early cold stress in the IR64 rice variety and further extended to different *indica* cultivars to understand the early signaling events associated with the cold stress response.

## Results

### Cold shock induces differential gene expression in IR64 *indica* rice

**Fig. 1:**
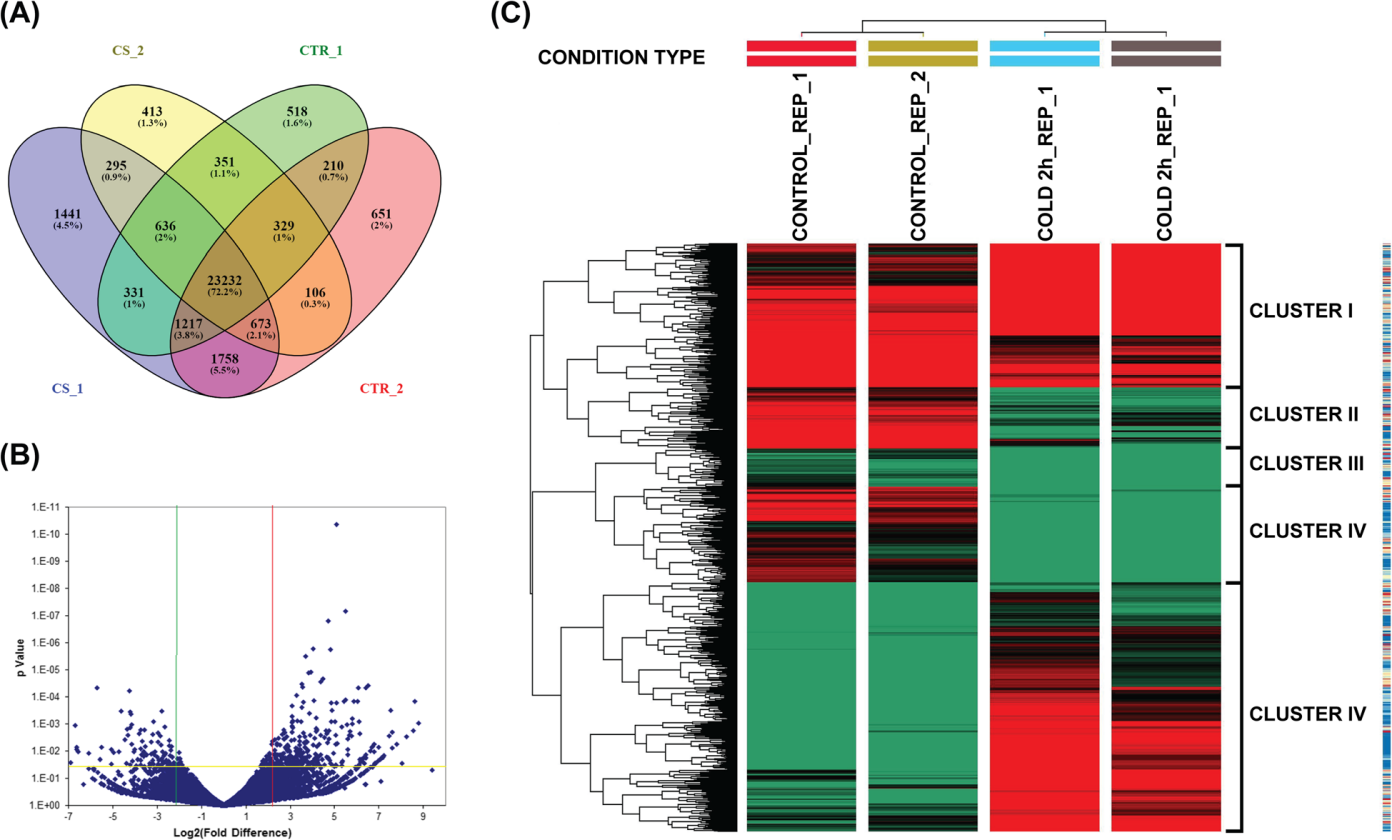
Differential Gene Expression in IR64 plants due to 2 h cold shock (**a**) Venn Diagram showing the distribution of the total 32,161 transcripts obtained in the RNA-seq replicates; The sets CTR1 and CTR2 represent the control replicates 1 and 2, whereas, CS1 and CS2 represent the Cold shock replicates 1 and 2 respectively. **b** Volcano plot showing the expression profile of the transcripts, the green and red lines indicate the log2 ratio cut-off for downregulated and upregulated DEGs, respectively. The yellow line represents the *p*-value cut off used for identifying the DEGs. **c** unsupervised hierarchical clustering of transcripts, with distinct upregulation and downregulation patterns in expression for cold shock replicates, compared to control condition. The count values are colour coded green to black to red in increasing order. Gene clusters exhibit classes of genes with distinct expression patterns under the two conditions

This work aimed at studying the early cold stress response in rice (*Oryza sativa* L. ssp. *indica*) involved the treatment of 14 days old rice seedlings to cold shock (at 4 °C) for 2 h. cDNA libraries generated from IR64 seedlings grown under the control (28 °C ± 1 °C) and 2 h cold shock (4 °C) conditions are denoted as CT replicates and CS replicates respectively. A total of 58.53 million (CT1), 33.61 million (CT2), 60.85 million (CS1), 39.82 million reads (CS2) were generated for the IR64 rice cultivars. Statistics of cleaned reads (approx. 63 nucleotides) were assessed with FastQC, which revealed that all reads were of fairly good quality and without adapters. The read mapping was carried out using HiSat2, resulting in 95.5% reads mapping to reference *Japonica* genome *Oryza sativa japonica* (Os-Nipponbare-Reference-IRGSP-1.0 (IRGSP-1.0) (Table 1). Normalized expression profiling was done on the aligned reads resulting in the identification of 32,161 transcripts expressed in at least one of the four samples profiled. As shown in Fig. 1a, 24,988 transcripts were found in both the control data sets, whereas 24,836 transcripts were common for both cold shock replicates. When compared between control and cold data sets, 72% (23232) of the transcripts were expressed in both, indicating a basal level of expression. Further, 539 transcripts were found exclusively expressed in control, whereas 931 transcripts were expressed only under cold shock condition.

**Table 1:** Statistics of IR64 transcriptome sequencing result from control and cold shock

**Parameter**	**5652_CONTROL (CTR1)**	**6767_CONTROL (CTR2)**	**5652_COLD 2 h (CS1)**	**6767_COLD 2 h (CS2)**
**Globals**				
Reference size	373,245,519	373,245,519	373,245,519	373,245,519
Number of Reads	58,532,485	33,609,748	60,853,958	39,822,202
Mapped paired reads	55,961,759 (95.61%)	32,135,283 (95.61%)	57,616,933 (94.68%)	38,452,860 (96.56%)
GC content	51.19%	52.59%	51.95%	52.62%
Unique transcripts	26,824	28,176	29,583	26,035
**Coverage**				
Mean	34.898	38.4396	32.9834	44.0307
Standard deviation	252.3602	217.7016	277.5779	295.2094
**Mapping**				
Mean Quality	23.42	20.12	23.75	19.95

We used Deseq2 package for differential expression analysis of the genes. A filter with a *p*-value cut-off of < 0.05 and log2fold change ≥1.5 and ≤ − 1.5 was set as the criteria to identify the differentially expressed genes (DEGs). These DEGs were visualized using volcano plot (Fig. 1b) to understand the distribution of up and downregulated genes. For this analysis, FPKM of > = 0.1 for a transcript was considered as expressed supported by a median read count of at least five reads per transcript covering 100% of the sequence. Among these DEGs, 380 genes were upregulated, whereas, 136 genes were downregulated in cold shock (CS) Vs control condition (CT) seedlings. (Additional file 1) Analysis of unsupervised hierarchical clustering of differentially expressed transcripts shows distinct gene expression patterns of up and down-regulation levels during cold shock treated (CS) (Fig. 1c). The differentially expressed genes could be categorized into five different clusters, based on their expression patterns. The largest group, Cluster V comprises of the differentially expressed genes, upregulated under cold shock, whereas, Clusters II and IV constitute the downregulated genes under cold shock. The clustering of all these replicates exhibited high sample reproducibility.

### Functional annotation of cold shock-induced genes indicate a significant increase in cold-responsive TF and ROS activity

**Fig. 2:**
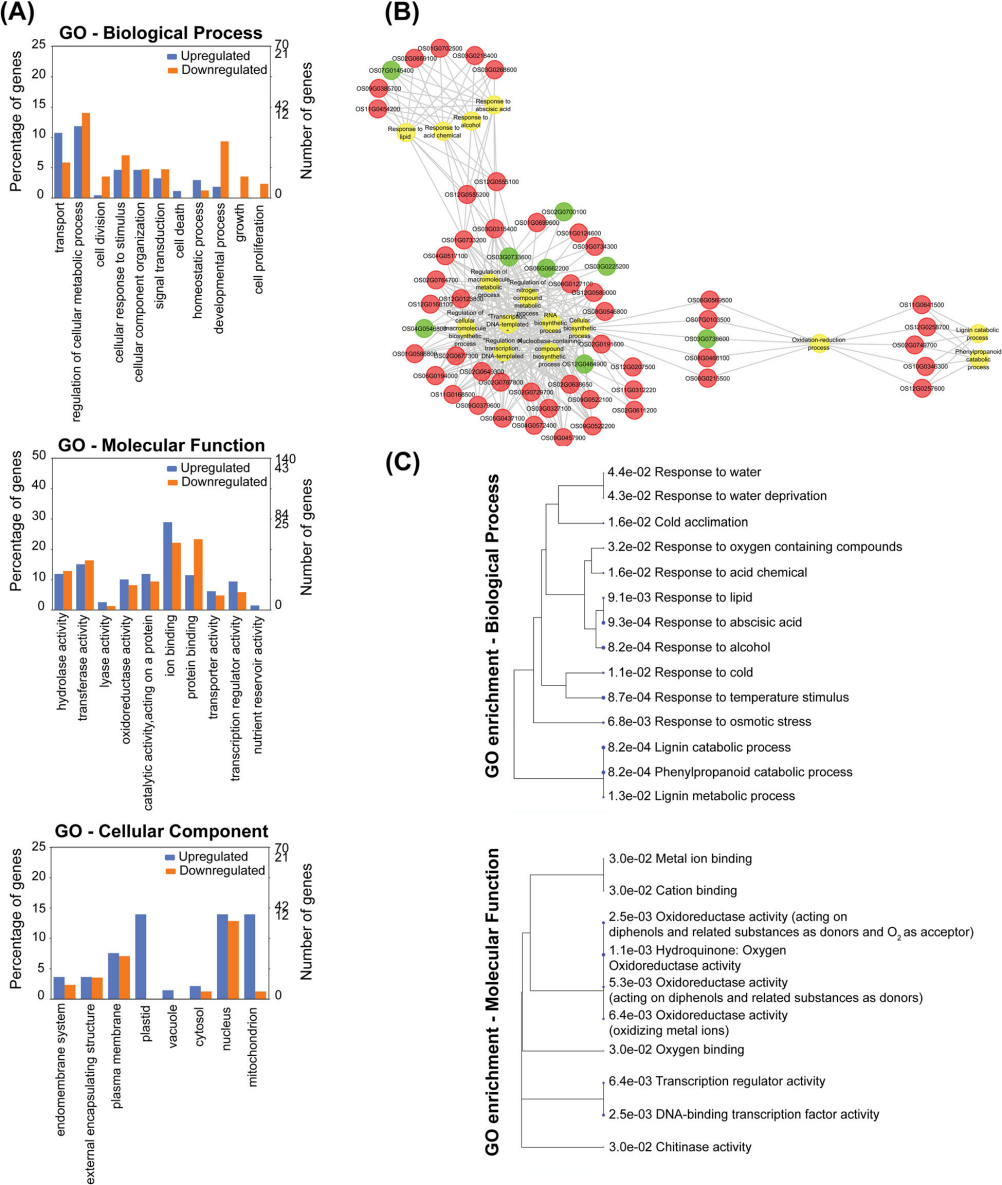
Gene ontology analysis of differentially enriched genes (**a**) shows the Histogram for gene ontology classification of upregulated (blue bars) Vs downregulated (orange bars) expressed genes. The results are summarized under GO categories: biological process, molecular function, and cellular component. **b** Differentially expressed gene enrichment map, obtained using Cytoscape, where the red and green circles represent the upregulated and downregulated loci and the yellow circles represent the enriched term. **c** Gene enrichment tree obtained for upregulated differentially expressed genes, with respect to their GO-Biological Process, and GO-Molecular Function; GO enrichment was performed using *Oryza sativa japonica* Group as the reference genome, with a p-value cut off (FDR) of 0.05

To understand the biological function of the cold induced differentially expressed genes (DEGs), GO enrichment was performed using an FDR adjusted *p*-value of ≤0.05 as the cut-off. The Blast2GO analysis for 516 DEGs featured 234 GO term annotation in biological process, 273 in molecular function (MF), and 262 for cellular component. Comparative analysis of the upregulated and downregulated GO terms indicates cell division (GO:0051301), proliferation (GO:0008283), developmental processes (GO:0032502) and growth (GO:0040007) were specific for downregulated genes. GO terms such as transport (GO:0006810), and homeostatic process (GO:0042592) were majorly associated with the upregulated genes (Fig. 2a).

GO analysis identified oxidation-reduction process, processes related to water stress and lignin metabolism were significantly enriched during cold shock treatment, in addition to generic terms such as cellular biosynthetic processes and transcription regulation (Fig. 2b). Under stress response, significant enrichment for GO-terms such as response to alcohol (GO:0097305), response to temperature stimulus (GO:0009266), response to abscisic acid (GO:0009737), response to lipid (GO:0033993), response to acid chemical (GO:0001101), response to osmotic stress (GO:0006970), and cold acclimation (GO:0009631) was observed (Fig. 2c). For the oxidation-reduction process, response to Oxygen-containing compound (GO: 1901700) and lignin catabolic process (GO:0046274) were significantly enriched under cold shock. GO-molecular function (MF) terms comparison indicates that metal ion binding (GO:0005488), oxidoreductase activity (GO:0016491) and transcription regulator activity (GO:0140100) were enriched for upregulated genes (Fig. 2c). GO enrichment analysis was also performed for the downregulated genes, but no significantly enriched terms were detected.

The biological pathway associated (KEGG pathway) with cold shock response was analyzed using BLASTKOALA (24.9% of input sequences). The analysis revealed that most genes were assigned to metabolism (40) of carbohydrate, amino acid, lipid and secondary metabolites; environmental information processing (15), genetic information processing (5) like transcription, translation and protein processes (Additional file 6C). Further, KEGG-BRITE reconstruction revealed that compared to control, a higher number of genes were assigned to ko01000 Enzymes (42), ko02000 Transporters (8), ko01003 Glycosyltransferases (6), ko03000 Transcription factors (4) and, ko04147 exosomes (4) in the cold shock treated sample (Additional file 2). The Interpro domain search data indicated that DNA binding domain and cytochromes were most abundant in the upregulated genes (Additional file 6B).

### Gene regulatory network induced during early cold stress

**Fig. 3:**
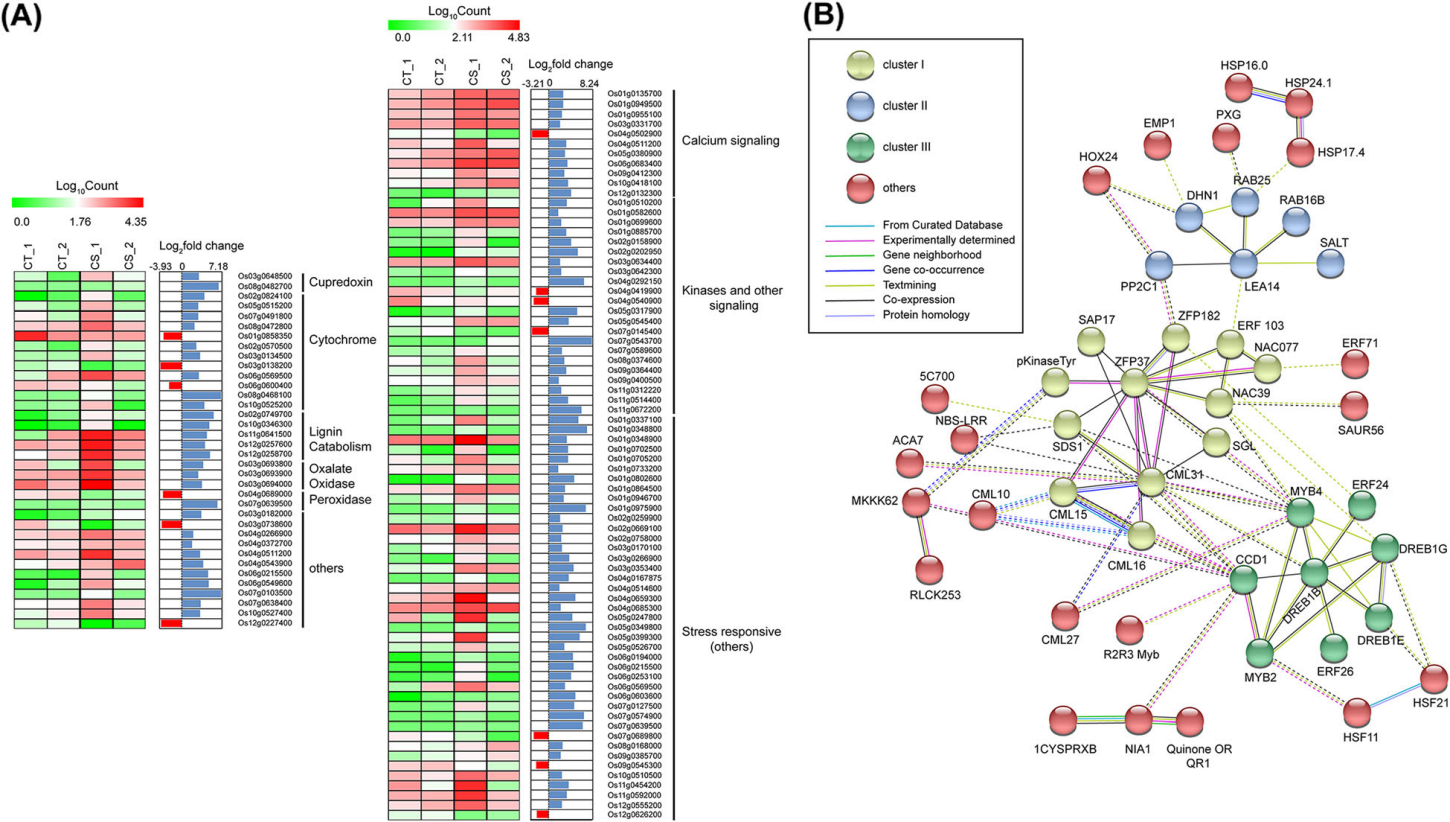
Differentially expressed stress-responsive genes under cold shock conditions**. a** Heat map generated using log_10_(count) values for each replicate, along with log2fold change obtained by DeSeq2 for Redox pathway components and other cold-responsive genes, respectively. **b** shows the interaction network of upregulated stress-responsive factors, obtained using the STRING database, with the minimum required interaction score of 0.400 and network edges representing evidence of an interaction. The legend for the colour of the nodes and edges are included in the figure

Analysis of the DEGs showed the presence of a milieu of stress responsive genes upregulated, which included heat shock protein genes (*Os02g0758000*, *Os03g0266900*, *Os06g0253100*) Terpene synthases (*OsTPS1*, *OsTPS31*), Dehydrins, LEA and RAB group of proteins coding genes (*OsDHN1*, *OsLEA28*, *OsRAB16*, *OsLEA14*), pathogen-related proteins and chitinase and glucanases. Among signaling molecules, calcium-calmodulin molecules and receptor kinases and protein phosphatases, along with several redox homeostasis proteins were induced under cold shock conditions. Besides hydrophilic proteins and signaling protein, our data set indicate the presence of 38 upregulated TFs (10% of total upregulated DEGs) and 9 downregulated TFs in cold shock transcriptome (Fig. 3a and b; Additional file 3). Gene network of these upregulated DEGs shows three major clusters that are highly interconnected. Cluster I comprise Zinc finger and NAC transcription factors and signaling proteins such as calmodulin and kinases. Cluster II represents DREB/AP2 and MYB transcription factors as major nodes. Cluster III contain proteins that mostly belong to osmoprotectants activated in response to dehydration stress (Fig. 3c).

#### Cell wall modification and ROS generation are crucial to stress perception during early cold shock in IR64

Differentially expressed gene set unique to this study (Additional file 4), has a significant number of genes responsible for cell wall modification and ROS generation (Fig. 3a and b). The genes coding for redox molecules comprised majorly of lignin catabolic laccase genes (*OsLAC10: Os02g0749700, OsLAC17: Os10g0346300, OsLAC23: Os11g0641500,* and *OsLAC29: Os12g0258700*), the germin-like oxalate oxidases, and other ROS generating enzymes. These findings suggest that the generation of ROS occurs during early cold shock and is essential for activating the redox signaling at the later stages of the stress response. Other stress responsive genes, unique to 2 h cold shock include the terpene biosynthesis genes (*OsTPS1*, *OsTPS10*), salt stress responsive lectin proteins (*Os01g0348800*, *Os01g0348900*) and receptor-like kinases (*Os11g0672200*, *Os04g0540900*). Other genes that were induced within 2 h include cell wall degrading enzymes, such as, chitinases (*Os05g0399300*, *Os11g0701200*, *Os11g0702100*), cellulases (*Os01g0946600*, *Os01g0946700*), pectin methylesterase (*Os04g0458900*), and a group of membrane transporter genes.

#### Cold shock-induced TFs upregulated under cold shock constitute major gene regulatory networks

**Fig. 4:**
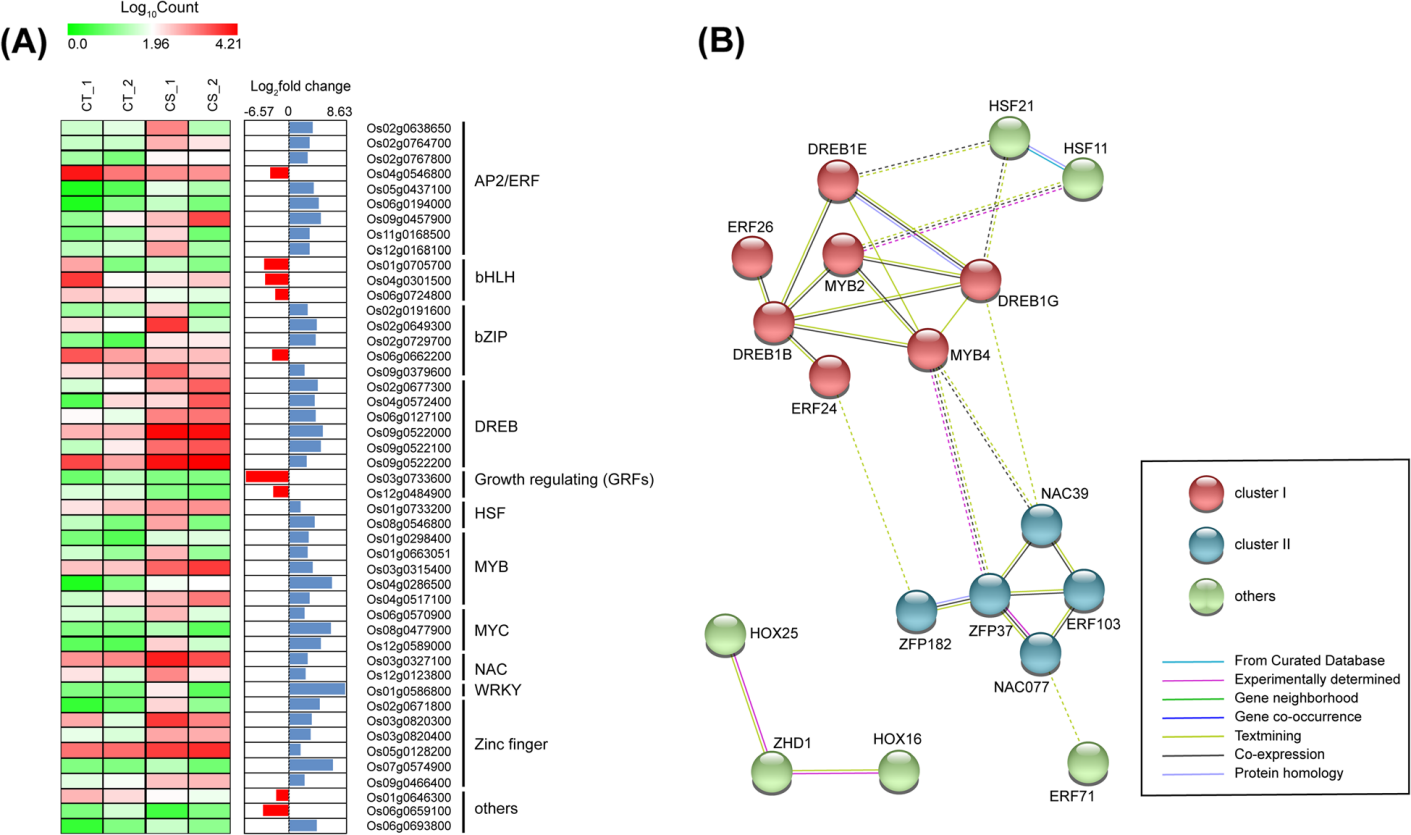
Differentially expressed transcription factors under cold shock conditions. **a** Heat map generated using log_10_(count) values for each replicate, along with log2fold change obtained by DeSeq2. **b** shows the interaction network of upregulated transcription factors, obtained using the STRING database, with the minimum required interaction score of 0.400 and network edges representing evidence of an interaction. The legend for the colour of the nodes and edges are included in the figure

Sequence analysis suggests that around 5–7% of coding sequences in plant genomes constitute transcription factors [37, 38]. In plants, the role of AP2/EREBP, bZIP, NAC/NAM (ATAF and CUC), MYC/MYB, and WRKY transcription factor families has been elucidated in the abiotic stress response that regulates stress-responsive gene expression via ABA-dependent or independent pathways [23, 39, 40]. The upregulated transcription factors in this study belong to various families; the majority being the AP2/ERF, DREB and MYB group of TFs (Fig. 4a). Downregulated genes included nine transcription factors needed for growth and development of the plant, which consisted primarily of bHLH TFs, and growth-related TFs such as *OsGIF3* (*Os03g0733600*), OsGRAS1(*Os01g0646300*) and *OsGRF7* (*Os12g0484900*) (Additional file 3). A gene regulatory network analysis using STRING shows that these upregulated transcription factors constitute a major network consisting of 15 nodes and a second network with 3 nodes. Further, the search suggested MYB2, MYB4, DREB1B, ZFP37, DREB1E and, DREB1G were highly connected and formed the central cluster (Cluster I, Fig. 4b). The cluster II consists of NAC39, which connects other TF like DERF5 (ERF103), NAC077 and ERF71. The heat shock transcription factors, HSF21, HSF11, are connected among themselves and also connected to central cluster via DREB1E, DREB1G and MYB2. HOX transcription factors constitute a secondary network which may contribute to cold stress response (Fig. 4b).

### Differential expression of DREB1 regulon genes is integral to early cold stress response

**Fig. 5:**
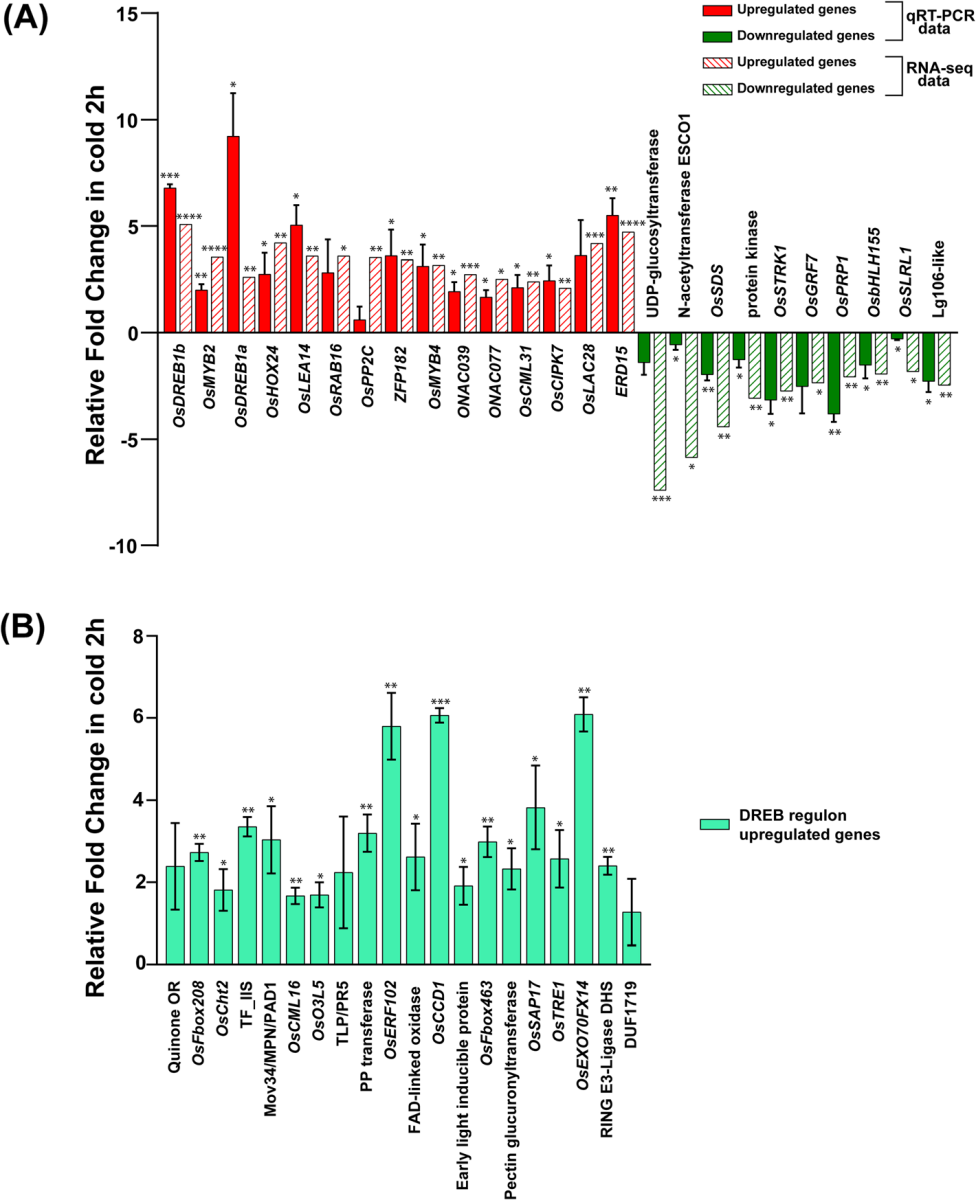
Validation of upregulated and downregulated DEGs in IR64 *indica* rice. **a** Validation of upregulated and downregulated genes using qRT-PCR. Histogram showing expression profiles of 15 upregulated (red bars) and 10 downregulated (green bars) genes, under 2 h cold shock (at 4 °C) conditions. Filled bars correspond to qRT-PCR data, and striped bars represent RNA-seq data, respectively. Error bar represents mean ± S.D. (*n* = 3; Two-tailed paired t-test, the level of significance was represented by * (where *P < 0.05 and > 0.0332, **P < 0.0332 and > 0.0021, ***P < 0.0021 and > 0.0002, ****P < 0.0001). **b** Validation of upregulated genes of the DREB regulon using qRT-PCR. Histogram showing expression profiles of 20 genes. Error bar represents mean ± S.D. (*n* = 3; Two-tailed paired t-test, the level of significance was represented by * (where *P < 0.05 and > 0.0332, **P < 0.0332 and > 0.0021, ***P < 0.0021 and > 0.0002, ****P < 0.0001)

The RNA-seq results from cold shock and control condition were validated using quantitative real-time PCR (qRT-PCR) for 25 genes. This included 15 genes from upregulated DEGs and 10 genes from downregulated DEGs of the cold shock transcriptome. Figure 5a shows the fold change obtained from three biological replicates for these 25 genes and their corresponding log2fold change obtained from RNA-seq data. As seen in the figure, 80% of validated genes exhibited differential expression with significant *p*-value (*p* < 0.05) that matches with the genes’ expression profiles from RNA-seq data.

In this study, we observed that the DREB1 group of genes viz., *OsDREB1A*, *OsDREB1B*, *OsDREB1C*, *OsDREB1G*, *OsDREB1E*, *OsDREB1H* were significantly upregulated under cold shock conditions. This indicates that the transcription factors which recognize the DRE motif are triggered early during cold shock, to induce downstream regulators for mounting the entire cold stress response in rice plants. Previous studies have indicated that DREB/CBF dependent regulation is considered as the major pathway in cold acclimation and is highly conserved in various plant species [41]. Among the 516 DEGs reported in this study, 27.7% of genes were identified to have at least one DRE-binding motif at the upstream region (Additional file 5). The upregulated DREB1 regulon genes identified in this study may be grouped into four major categories: the hydrophilic proteins, LEA, DHN1 (COR410) proteins, stress associated proteins (SAP17), osmotin precursors, photoperiod sensitive and transporter proteins; signal transducing molecules, such as phosphatases, membrane kinases, Ca^2+^-CaM proteins, and various cold-responsive ubiquitin ligases; multiple classes of enzymes including catabolic chitinases, exocyst complex proteins and oxidoreductases, laccases; and the final group of other zinc fingers, HOX, and AP2/ERF group of transcription factors. Twenty genes from the 107 upregulated DREB regulon genes were validated, among which expression of the genes coding for AP2/ERF transcription factor *OsERF102* (*Os09g0457900*), Calcium-binding protein *OsCCD1* (*Os06g0683400*), and the exocyst subunit EXO70 family protein, *OsEXO70FX14* (*Os01g0905300*) exhibited high levels of significant upregulation (Fig. 5b).

### Expression of cold-responsive genes in different unexplored *indica* cultivars

**Fig. 6:**
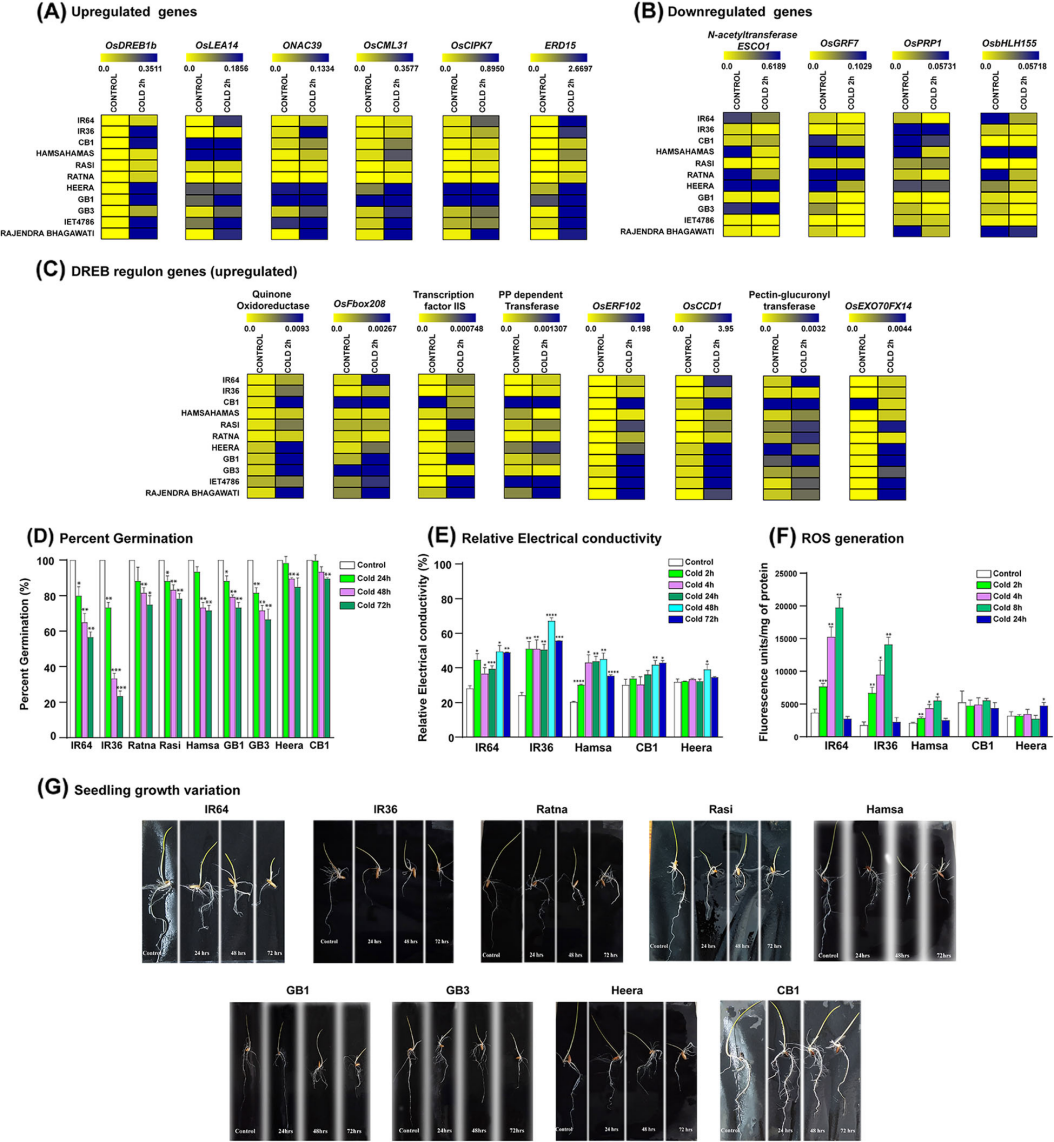
Expression of cold-responsive genes and physiological responses in different *indica* rice. **a**-**c** Heat maps show the qRT-PCR results of 18 differentially expressed genes in different *indica* rice varieties. The 2^-dCT values (expression profile) are plotted for upregulated genes (**a**) downregulated genes (**b**), and upregulated DREB regulon genes (**c**) respectively, under control and cold shock conditions. **d** Histogram representing percent germination of nine different *indica* rice varieties **e**, **f** Histogram representing the data obtained from physiological response experiments for IR64, IR36, Hamsahamas, CB1, and Heera *indica* rice varieties. **d** Histogram showing Relative Electrical Conductivity percent (REC%) for the five rice varieties. **f** Histogram showing the ROS generation expressed as fluorescence units/mg of protein. **d**-**f** In each case, the experiment was repeated in triplicate sets. The bar values are expressed as mean ± S.D. (*n* = 3; Two-tailed paired t-test, the level of significance was represented by * (where *P < 0.05 and > 0.0332, **P < 0.0332 and > 0.0021, ***P < 0.0021 and > 0.0002, ****P < 0.0001). **g** Images showing germination of different *indica* varieties post cold stress. Image was taken 7 days post germination

We extended our study to ten different *indica* rice cultivars that were never characterized for cold stress response. These varieties are mostly high yielding varieties (HYVs), including both hybrids and field-selected varieties, cultivated all over India (Table 2). The expression profiles of 18 genes (14 upregulated and 4 downregulated, as validated in IR64) from the above-validated list were tested for this study. Our data shows upregulation of transcript levels of *OsDREB1b*, *ONAC039*, *OsCML31*, *ERD15*, *OsLEA14, OsCIPK7* and the DREB regulon genes Quinone Oxidoreductase, *OsFbox208*, Transcription factor IIS, Pyridoxal phosphate dependent transferase, *OsERF102*, *OsCCD1*, Pectin-glucuronyltransferase, and *OsEXO70FX14* genes post cold shock treatment in the majority of the varieties (Fig. 6a). In the case of the downregulated genes, *OsGRF7*, *OsPRP1*, *OsbHLH155* showed consistent down-regulation in all varieties in response to cold shock treatment (Fig. 6b). Our data indicated that expression of majority of the cold-responsive genes was significantly upregulated in the CB1, and Heera cultivars under control conditions, compared to the other varieties. Interestingly, the DREB regulon genes *OsEXO70FX14* (*Os01g0905300*), Transcription Factor IIS (*Os06g0693800*), Pyridoxal-phosphate dependent transferase (*Os04g0614500*), *OsFbox208* (*Os04g0414500*) which exhibited highly significant upregulation under cold shock conditions in most varieties, was highly upregulated under control condition in CB1 rice variety (Fig. 6c). This higher level of expression of cold-responsive genes under control condition in CB1 and Heera varieties prompted us to examine the physiological response of the indica rice varieties used in this study.

**Table 2:** List of *indica* rice varieties used in this study

**SL**	**Name of Variety**	**Parentage**	**Year of Notification**	**Growth Duration (in days)**	**Eco-System**
1	Hamsahamas	Traditional rice variety of Bengal		130–135	Rainfed Upland
2	Ratna (IET-1411)	TKM-6 x IR-8	1974–78	130–135	Upland and direct seeded
3	Rasi (IET-1444)	T(N)1 x Co.29	1978–82	120–125	Rain fed Upland Areas
4	IR-36 (IET-4555)	IR-8 x Tadukan x TKM-62 x T(N)1 x IR-243 x *Oryza nivara*-4 x IR-8 x PTB-21 & PTB-18	1982	112–115	Irrigated, Rainfed Upland or Lowland
5	IR-64(IET-9671)	IR-5857-33-2-1 x IR-2061-465-1/5/2005	1991	115–120	Irrigated Areas
6	Gontra Bidhan 1 (GB1) (IET-17430)	Selection from the farmers’ field	2008	118	Irrigated (Early & Mid. Early)
7	CB1	Selection from boro germplasm collected in Hooghly district of WB	1952	120–130	Upland
8	Rajendra Bhagwati	RAU 1397–18–3-7-9-4-2	2010	110–115	Upland/ Midland
9	Satabdi (IET-4786) (Miniket)	CR-10-114 x CR-10115	2000	112–115	Irrigated Medium
10	Heera	CR-404-48 x Cr-289-1208	1989	110–120	Rainfed Upland
11	Gontra Bidhan 3 (GB3) (IET-22752)	Selection from the farmers’ field	2012	120–130	Irrigated and rainfed upland

The germination experiments performed in this study revealed that the *indica* rice variety CB1 remains nearly unaffected by cold stress (72 h) prior to germination, whereas, the variety IR36 shows the highest sensitivity to the same. As illustrated by Fig. 6d, the decrease in percent germination of CB1 with increasing cold stress is not significant, compared to the other varieties. The rice variety Heera also exhibits a slight reduction in the percent germination, which indicates that this variety may also have better resilience to the low-temperature stress. IR64 and IR36 varieties show a significant drop in their percent germination, which suggests their high level of sensitivity to cold stress conditions. All the other *indica* varieties used in this study exhibit low to moderate sensitivity to cold stress as shown by their percent germination (Fig. 6d).

The electrolyte leakage of the *indica* rice varieties IR64, IR36, Hamsahamas, CB1, and Heera under control and cold stress conditions (4 °C treatment for 2 h, 4 h, 24 h, 48 h, and 72 h) was studied by determining the relative electrical conductivity (REC%) of leaf tissue samples of 14 days old seedlings. As shown in Fig. 6e, REC% shows a significant increase under cold stress in IR64, IR36, and Hamsahamas varieties. In contrast, a delayed increase of REC% was observed for CB1, and Heera varieties (48 h and 72 h cold stress treatments). There was no significant change in REC% in CB1 and Heera during initial cold stress time points.

The ROS generation of the five indica varieties, under control and cold stress conditions (4 °C treatment for 2 h, 4 h, 8 h, and 24 h) was quantitated using H_2_DCFDA. Our data shows that the ROS generation is induced significantly under cold shock conditions in IR64, IR36 and Hamsahamas varieties, with higher levels of increase in IR64 and IR36 varieties. As exhibited in Fig. 6f, the ROS levels in these three varieties show a significant increase with increasing stress duration, up to 8 h. However, the ROS generation decreases after 24 h of cold treatment in IR64, IR36 and Hamsahamas, suggesting the induction of the ROS scavengers during later stages of cold stress. The tolerant varieties CB1, and Heera show no significant changes in the ROS levels during cold stress.

## Discussion

Studies have implicated cold stress as a major threat for *indica* rice plants in upland areas. The low temperature of the water used for irrigation in such area results in a lower rate of germination, delayed seedling emergence, increased electrolytic leakage, changes in chlorophyll fluorescence, retarded growth, lower spikelet fertility, reduced tillering and high death rate [1–4]. Therefore, despite many high yielding varieties (HYVs) being developed, the challenge remains to grow such HYVs under low-temperature conditions.

This study was designed to identify the early changes in the expression associated with cold stress response in IR64 *indica* rice variety. Functional annotation of upregulated DEGs generated from this study exhibited a high level of significant enrichment for GO-terms, such as transcription factor activity, response to temperature stimulus, response to osmotic stress, and response to redox changes. Analysis of genes unique to this study is centralized around stress perception and early signal relays, such as altered membrane rigidity and electrolytic leakage, the onset of calcium signaling, ROS generation and activation of stress responsive transcription factors. Interestingly, when compared with previous reports of cold stress responsive genes in *indica* varieties, IR64 early cold response is associated with a higher number of upregulated genes unlike late chilling stress response in 93–11 variety (4 °C for 72 h), (2298 upregulated and 3992 downregulated DEGs, [42]).

The role of transcription factors in stress response has been extensively studied in rice plants. Reports suggest that manipulating the expression levels of various stress induced TFs alters the expression of downstream target genes that are involved in enhancing stress tolerance mechanism in plants. In rice, while the role of WRKY family of transcription factors has been more prevalent in plant-pathogen response; AP2/EREBP and NAC/NAMTFs have gained more considerable significance in abiotic stress response studies [43–45]. Our data elucidates the upregulation of 38 transcription factors (10% of total upregulated DEGs) in the cold transcriptome, where AP2/EREBP family represent the dominant group. The role of DREB1 genes, a subtype of AP2/EREBP TF family, in cold stress response via the ABA-independent pathway has been well established in rice plants [4, 46–48]. Studies have reported that *OsDREB1A* and *OsDREB1B* gene expression was induced within 40 min after cold exposure [40]. Interestingly, our results show similar upregulation of the DREB1 genes: *OsDREB1B*, *OsDREB1A*, *OsDREB1C*, *OsDREB1E*, *OsDREB1G*, and *OsDREB1H* genes in *indica* rice IR64 within 2 h of cold shock. A large number of other AP2/ERF transcription factors, such as *OsERF141*, *OsDERF5*, *EREBP139*, *OsERF102*, and *OsDERF8* were also upregulated; further indicating the importance of the AP2/EREBP TFs in early cold stress response [49]. The R2R3-type Myb TF in rice, *OsMYB2* has been reported to be a master regulator for abiotic stress response conferring salt, cold and dehydration stress tolerance when overexpressed in rice [50]. Overexpression of *OsMYB4* has imparted significant tolerance to chilling and freezing stress in transgenic *Arabidopsis* [51–53]. Furthermore, Dai et al. [54], suggested that the R1R2R3 Myb factor, OsMYB3R-2 was upregulated under cold stress (72 h) and activated DREB/CBF pathway to increase the tolerance of the plant against freezing, drought and salinity stress [55]. Our study also indicates upregulation of *OsMYB2* and *OsMYB4* genes within 2 h of cold shock, thus implying their involvement in early signaling events. ZFP182 (*Os03g0820300*) gene, coding for a TFIIIA-type zinc finger protein type transcription factor known to be involved in multiple abiotic stress tolerance mechanisms in rice [56, 57] was significantly upregulated under cold 2 h shock in this study (Additional file 3).

Sensing of the low temperature occurs via changes in the membrane fluidity or by sensor proteins such as RLKs, phospholipases, and Calcium channels. This perception leads to cytoskeletal reorganization due to Ca^2+^ influx, triggering several signaling events, thereby mounting the entire cellular response during stress [4, 58, 59]. The signaling proteins upregulated in this study involves components in calcium signaling such as Calmodulin/CBL, Ca^2+^ transporter and Ca^2+^ decoder & various other kinases, such as RLKs, RLCK, MAPKKK and Phospholipase A2 (Additional file 3). The role of these proteins is to transduce the cold signal in the cytosol and activate stress responsive transcription factors and various hydrophilic polypeptides such as LEA and Dehydrin, to stabilize against cold-stress injuries in plant cells. Increased electrolytic leakage in IR64 rice variety was evident from a significant increase in the relative electrical conductivity (REC%) for plants subjected to cold 2 h stress treatment at 4 °C (Fig. 6e).

The Late Embryogenesis Abundant (LEA) group of proteins, as the name suggests, are synthesized at the later stage of embryogenesis, prior to seed desiccation [60]. Reports in barley, indicate that the expression of LEA genes is induced under higher ABA concentration and water-deficit conditions, asserting their role as a dehydration responsive gene [61]. Several LEA, dehydrins and low temperature-induced [62] genes were upregulated in our data set (Additional file 3), suggesting the onset of dehydration response during initial cold stress conditions. Among this, *OsLEA14* or *wsi18* which was previously reported to have a role in chilling stress [63, 64] was also found to be present as upregulated LEA under 2 h cold shock condition.

Low-temperature stress triggers the generation of ROS like singlet oxygen species and H_2_O_2_ [65]. This is metabolized by ROS scavenger like cytochrome P450 to prevent cellular damages, ultimately leading to redox homeostasis. Studies suggest that production of ROS varies during abiotic stress response between sensitive and tolerant varieties of rice [66–68]. Zhang et al. have shown that rice can better adapt to the chilling stress condition when ROS-mediated signaling genes were upregulated. Our result shows upregulation of *Os71Z6*, *CYP78A9*, *OsKO4*, *OsABAox2*, *CYP701A8*, and *OsNR1* genes of the cytochrome family in response to cold shock. Reports suggest that the germin-like-oxalate oxidases that generate H_2_O_2_ in the apoplast [69] may have a role in plant defense response. Upregulation of these oxalate oxidases during cold stress response in our study agrees with the finding that ROS generation is induced as a primary abiotic stress response [70]. Further, the significant increase in ROS production was observed under cold shock condition in IR64 variety, as observed by increased H_2_DCFDA fluorescence for cold 2 h treated plants as opposed to control plants (Fig. 6f). Similar to the observations from Zhang et al. study, our data shows pathway enrichment of Plant-Pathogen interaction and Diterpenoid Biosynthesis (adjusted *p*-values < 0.014 and 0.011 respectively) when IR64 plants were subjected to cold shock conditions (Additional file 6A). Studies in *Arabidopsis* report that Aquaporins or TIP1 and TIP2 are required for the transport of H_2_O_2_ species from the chloroplast and peroxisomes to the cytoplasm for regulating ROS signaling [71]. Interestingly, our results show upregulation of both ROS transporter Aquaporin (*OsTIP1*) and heat shock transcription factors (HSFs) as molecular sensors of ROS, in early cold stress response to regulate the oxidation stress responsive genes [72] (Additional file 5). Various other cold stress response genes established by previous studies, such as *OsTPP2* [73], *OsTPS1* [74], *OsPHS1* [75], were significantly upregulated during cold 2 h shock in this study.

The Dehydration Responsive Element (DRE) cis-acting elements are involved in both osmotic and cold stress induced gene expression. While osmotic stress response involves changes in DREB2 regulon gene expression, DREB1 genes are majorly responsible for mounting the cold stress response in plants [76]. Further, several studies have reported that overexpression of the *DREB1A*, *DREB1B*, and *DREB1C* genes lead to certain biochemical alterations that are associated with the phenomenon of cold acclimation, and freezing stress tolerance in *Arabidopsis* [77–81]. Reports suggest that on perceiving the cold stress signal, the DREB1 cassette genes are promptly and transiently expressed, which then activates a milieu of downstream stress responsive genes in both dicotyledons and monocotyledons [82]. In this study, significant upregulation of the DREB1 gene cassette prompted us to focus on the DREB regulon genes. Genes with the A/GCCGAC (DRE core motif) site in their 1 kb upstream sequences were screened from among the differentially expressed gene set. Upregulated DREB regulons (107 genes), obtained from this analysis include abiotic stress responsive TFs, calcium-binding proteins, RLCKs, redox- signaling molecules, and other hydrophilic water-deficit responsive proteins. The down-regulation of certain DREB regulons (36 genes) during cold shock suggests that they may be associated with normal growth and development of the plant (Additional file 5).

Cold stress, like any other abiotic stress, has an adverse effect on normal growth and metabolism in rice plants. The validated genes from the downregulated differentially expressed list are mostly related to cell cycle, protein kinases, and growth-promoting transcription factors. UDP-glucosyltransferase N-acetyltransferase (*Os01g0686300*) and Protein kinase (*Os07g0145400*) are genes encoding proteins involved in metabolic processes, such as transferring of hexosyl groups, and phosphate, respectively. These genes were significantly downregulated under cold shock, indicating hindrance to metabolism in rice plants. N-acetyltransferase ESCO1 (*Os04g0498900*) which codes for an acetyltransferase having a role in meiotic chromosome segregation and sister chromatid cohesion was found to be downregulated. This suggests that under cold shock inhibition of chromosomal segregation occurs that may lead to inhibition of the cell cycle. Cell cycle genes such as *OsSDS* (*Os03g0225200*) that codes for Cyclin A/B/C/D domain-containing protein and endosulphine family protein Lg106 (*Os01g0249300*) involved in the initiation of G0 program were both downregulated; further emphasizing the negative effect of cold stress on the cell cycle. Transcription factors significantly downregulated under cold shock included Basic helix-loop-helix dimerization region bHLH domain-containing protein, *OsbHLH155* (*Os06g0724800*) and Transcription factor-GRAS domain-containing *OsSLRL1*(*Os01g0646300*), responsible for gibberellin (GA) signaling in plant growth and development. Transcription activator, *OsGRF7* (*Os12g0484900*), growth-regulating factor 7, responsible for growth and development, was also downregulated under cold shock condition.

Germination experiments were employed to identify *indica* rice varieties with contrasting physiological response to cold stress. CB1 and Heera showed better germination profile under cold treatment, which was highly contrasted to the sensitive varieties, IR36 and IR64 exhibiting reduced rates of germination under cold stress condition (Fig. 6d and g). These varieties, along with one with semi-cold sensitive variety Hamsahamas were then subjected to relative electrical conductivity and ROS generation studies. IR64 and IR36 rice varieties exhibited a significant increase in REC% and ROS production under cold shock conditions. This indicated that electrolytic leakage and ROS generation are triggered early during cold stress response in the sensitive rice varieties, as opposed to the tolerant lines, CB1 and Heera. The low level of REC% and ROS production, together with the expression profile of the cold-responsive genes in CB1 and Heera rice varieties further validate their high level of tolerance to the low-temperature condition (Fig. 6e and f). Furthermore, transcription of some of the cold stress responsive genes and cold induced DREB regulons were found to be higher in CB1 and Heera varieties under control conditions suggesting that these varieties may be primed to mount the stress response during cold shock. However, all the DEGs that were profiled in other *indica* varieties show upregulation under cold shock condition (Fig. 6a to c).

Studying the cold induced change in transcription may be one approach to decipher the complexity of cold signaling in plants. However, deciphering the relationship between gene expression and dynamicity of the epigenome, changes in metabolites, alteration in protein modifications together, can lead to a better understanding of signaling network associated with cold stress response especially in cereal plants like rice.

## Conclusions

This study was aimed at identifying some of the key responsive genes activated early under cold stress (2 h) in IR64 *indica* rice variety. The extensively studied cold-responsive DREB subfamily members were induced as an early event, along with other EREBP, MYB, NAC, HSF transcription factors families. A large number of Ca^2+^ binding proteins and kinases indicate that calcium signalling as an essential cellular mediator of the cold signal perception and response. Triggering of the ROS generation is also evident as numerous ROS producing oxidases were upregulated in the cold shock transcriptome. The growth promoter factors and transcription factors were downregulated as an initial response to the cold condition. Taken together, this study indicates that the Ca^2+^ and ROS mediated pathways are early cold shock-induced events which prime the cells for the later response. Physiological and expression studies with different *indica* rice cultivars suggest that CB1 and Heera *indica* rice varieties are better suited for low-temperature conditions, as opposed to the highly sensitive IR36 and IR64 varieties.

## Methods

### Plant growth conditions

*Oryza sativa* L. ssp. *indica* rice genotypes used in this study include IR64, CB1, Heera, Hamsahamas, Ratna, Rasi, IR36, GB1, GB3, IET4786 (Miniket), and Rajendra Bhagawati (RB) (Table 2). The seeds were surface sterilized with 0.1% (w/v) HgCl_2_ for 15 min, washed several times with sterile water, following which they were germinated over water-soaked sterile gauge placed in trays at 28 °C ± 1 °C in the dark for 3 days. The germinated seedlings were transferred to fresh water-soaked sterile gauge in trays, in the presence of 0.25X Murashige and Skoog complete media at 28 °C ± 1 °C in 16 h light and 8 h dark photoperiodic cycle with 50% relative humidity and 700 lmol photons m^− 2^ s^− 1^ in a plant growth chamber. For cold shock treatment, the 14-days-old seedlings (~ 100 seedlings for each experimental set) were transferred to 4 °C, whereas the control plants (~ 100 seedlings) were maintained at 28 °C ± 1 °C (marked as CS and CT respectively). For cold shock, seedling samples were collected from plants incubated at 4 °C for 2 h.

### RNA extraction

RNA was isolated from ~ 200 mg of leaf tissue of rice seedlings (n ~ 6 seedlings) for qRT-PCR from each treatment (control, CT and cold shock, CS) of IR64 cultivar (considered as a single replicate). Total RNA from each replicate was extracted using RNASure® Mini Kit (Nucleopore-Genetix), according to manufacturer’s protocol. RNA samples were treated with DNaseI to remove DNA contamination. For RNA-seq, RNA was pooled from ~ 500 mg seedlings (n ~ 15seedlings) and considered as one biological replicate. Two such replicates for each control and cold shock (CS) samples were used for sequencing. The RNA concentration was determined using the Qubit Fluorometer. An aliquot of the samples was run on an (Agilent) RNA Bioanalyzer chip to check for integrity.

### cDNA library preparation and Illumina sequencing

The RNA quality check, quantification, cDNA library preparation and sequencing were done at Genotypic Technology, India using Illumina NextSeq500 platform generating 400 million paired-end reads with an average size of 75 bp.

### Raw sequence processing and differential gene expression

Raw reads were assessed using FastQC [83]. The adapters attached with the raw reads were subsequently removed using BBDuK [84] tool and quality was confirmed using FastQC. Processed reads were corrected using Rcorrector [85]. HiSat2 index file was built using *japonica* reference genome and gtf files (gene transfer format) containing the feature list from RapDB (https://rapdb.dna.affrc.go.jp/download/archive/irgsp1/IRGSP-1.0_genome.fasta.gz) and (https://rapdb.dna.affrc.go.jp/download/archive/irgsp1/IRGSP-1.0_representative_2019-03-22.tar.gz). Better and complete annotation of the *Japonica* rice was the rationale behind using it as the reference genome [86]. HiSat2 [87] was used to align corrected reads with the reference genome. The alignment output file was in SAM format and was converted into BAM format using SAMtools [88]. Qualimap [89] analysis was done to assess the quality of alignment on sorted BAM files. FeatureCounts [90] was used to convert sorted BAM file to count reads. Later, DeSeq2 [91] that takes count files as input was used for calculating differentially expressed genes. In order to filter out differentially expressed genes, threshold fold-change was set at 1.5 with a *p*-value cut off of ≤0.05. Subsequently, unsupervised hierarchical clustering and analysis were done on analyzed output.

### Functional annotation

The BLAST2GO [92, 93] program was used against NR database for the GO annotation analysis of DEGs (Differentially Expressed Genes) in terms of Biological Process, Molecular Function and Cellular Components ontologies. WEGO tool was used to visualize the GO annotation, where both the upregulated and downregulated GO data sets were uploaded to obtain a comparative GO analysis. KO (KEGG Orthology) analysis was performed by submitting the upregulated gene list in BLASTKOALA [94]. PANTHER [95] classification system was used to determine the protein family classification. Significantly enriched GO and KEGG Pathways of the differentially expressed genes was carried out using ShinyGO v0.60 [96] online tool and Cluster profiler package [97].

Further, biologically relevant and statistically significant enriched genes were identified, and regulatory network modelling analysis was carried out using in-house scripts, and the output was visualized using Cytoscape V2.8.3 [98]. The p-value calculated by the hypergeometric test and was corrected by FDR. The FDR value of ≤0.05 was used as the threshold to identify the significant functional categories and metabolic pathways. Upregulated genes including transcription factors, kinases, calcium signaling components, redox components and other stress responsive genes were filtered out from the upregulated gene list and submitted to STRING [99] database for network analysis.

### DRE motif searching in upstream of genes

To search for the DRE (Dehydration Responsive Element) motifs, DNA sequences were fetched within 1 KB upstream (from the transcription start site) of the coding genes using getfasta program (bedtools) [100]. The extracted region sequences of the differentially regulated genes were and examined for the DRE core-motif (A/GCCGAC) [101]. The Find Individual Motif Occurrences (FIMO) tool of the MEME package was used for motif search [102].

### qRT-PCR

For qRT-PCR, RNA was isolated from different samples using TRIzol reagent (Invitrogen) as described in manufacturers’ protocol. cDNA samples were generated using 5 μg of total RNA from three biological replicates. The cDNA thus generated was used for subsequent validation experiments. Forty-five genes (35 upregulated and 10 downregulated) were selected from the differentially regulated gene list, based on the potential role in cold stress response, presence of DRE sites and literature study. The primers used for this study are listed in Additional file 7. *OsActin1*(*Os03g0718100*) and *OsUbq5* (*Os01g0328400*) genes were selected as endogenous control genes. All reactions were performed in three independent biological replicates, and the expression levels for each sample were calculated using the ΔCt method. Two-tailed paired t-test was performed to determine the level of significance. In case of the expression studies in different *indica* varieties (Fig. 6a to c), the 2^-ΔCt^ values were used to generate heatmaps for each gene, under control and 2 h cold shock conditions.

### Percent germination under cold stress condition

Surface sterilized seeds of the nine *indica* rice varieties (IR64, IR36, Hamsahamas, Rasi, Ratna, Heera, CB1, GB1, and GB3) were incubated at 4 °C for varying time points: 24 h, 48 h, and 72 h under moist conditions. Following each cold stress time points, the seeds were brought to 28 °C ± 1 °C and incubated for 3 days to mimic the control germination condition. Finally, ten seeds of each variety were plated in separate sterile Petri plates on adequately moist 90 mm filter paper disc. The germination rate, root length, and shoot length of each seed was recorded for the next 5 days. Three biological replicates were generated for each variety, and two-tailed paired t-test was performed to determine the level of significance. The percent germination of the varieties was calculated as under:

$$ Percent\ germination=\frac{number\ of\ germinated\ seeds}{total\ number\ of\ seeds}\times 100\% $$

### Determining the electrolyte leakage (using REC assay) and ROS production in *indica* varieties

The relative electrical conductivity (REC) of five *indica* rice varieties (IR36, IR64, Hamsahamas, CB1, and Heera) was measured under cold stress condition, to determine the variation in the physiological response of these varieties. For this, 14 days old seedlings of each variety were subjected to 4 °C treatment for 2 h, 4 h, 6 h, 24 h, 48 h, and 72 h respectively. Seedlings maintained at 28 °C ± 1 °C were used as the control set. Two hundred milligrams of leaf tissue from each set (after stress treatment, and control) was harvested and immersed in tubes containing 20 mL of distilled water. The tubes were then incubated at room temperature (25 °C) with constant shaking at 120 rpm [42] for 2 h. After incubation, the initial electrical conductivity of the solution (EC1) was measured, which represents the ion leakage from the leaf tissue samples. Following measurement of EC1, the solution was heated to a temperature of 100 °C for 30mins, cooled to room temperature, and the electrical conductivity of the solution (EC2) was measured at room temperature. The electrical conductivity of distilled water, EC_w_1 and EC_w_2 were measured for normalization purpose [103]. The relative electrolyte content (REC) was calculated as under:

$$ REC\ \left(\%\right)=\left[\frac{EC1- ECw1}{EC2- ECw2}\right]\times 100\% $$

The ROS production of IR36, IR64, Hamsahamas, CB1, and Heera *indica* rice varieties was determined under control and cold stress treatment (at 4 °C for 2 h, 4 h, 8 h, and 24 h) conditions. Ground tissue powder from 100 mg leaf tissue samples of 14 days old seedlings of each variety was suspended in 10 mM Tris-Cl (pH = 7.2). After removing the cellular debris, the plant extract (was diluted?) was subjected to ROS measurement using H_2_DCFDA (2′7’- Dichlorofluorescein diacetate: 100 mM solution in DMSO). The protein concentration of each sample was determined using the Bradford reagent. BSA standard curve was plotted to determine the protein concentration. The ROS generation of the samples was expressed as fluorescence units/mg of protein [104].

For both the physiological experiments, three biological replicates were generated for each variety, and two-tailed paired t-test was performed to determine the level of significance.

## Supplementary information

**Additional file 1.** Table containing detailed analysis of cold shock (2 h at 4 °C) significantly upregulated and downregulated genes, compared to control conditions, in IR64 seedlings.

**Additional file 2.** Sheet 1 contains a table for GO enrichment, genes for each functional category for upregulated DEGs. Sheet 2 contains a table showing KEGG Brite analysis for cold induced DEGs.

**Additional file 3.** Gene details for differentially regulated Transcription factors, components of Redox pathway, and other cold-responsive genes.

**Additional file 4.** Differentially expressed genes unique to IR64 cold shock 2 h transcriptome data.

**Additional file 5.** Differentially regulated gene list with DRE-core motif in 1 kb upstream of the transcription start site.

**Additional file 6.** Functional annotation for differentially regulated genes. (A) KEGG pathway enrichment data for upregulated DEGs (B) Top ten abundant domains present in the upregulated genes, obtained using Blast2GO (C) the pathway reconstruction result of upregulated differentially expressed genes, based on KEGG, generated using BLASTKOALA.

**Additional file 7.** Table containing sequences of primers used for qRT-PCR assays.)

## Data Availability

The datasets generated and/or analysed during this current study are available in the NCBI Sequence Read Archive repository (https://www.ncbi.nlm.nih.gov/bioproject/?term=PRJNA506503) under the following accession numbers: SRX5055383 (IR64_cold 2 h_replicate_1), SRX5055384 (IR64_cold 2 h_replicate_2), SRX5055385 (IR64_control_replicate_1), and SRX5055386 (IR64_control_replicate_2).

## Abbreviations

CT: Control seedlings
CS: Cold shock treated seedlings
DEGs: Differentially expressed genes
DRE: Dehydration responsive element
FIMO: Find Individual Motif Occurrences
GO: Gene Ontology
HYVs: High yielding varieties
KO: KEGG Orthology
ROS: Reactive oxygen species
TFs: Transcription factors
qRT-PCR: Quantitative real-time PCR
H_2_DCFDA: 2′,7′-dichlorodihydrofluorescein diacetate
