# Supplementary figures and images for: Understanding the early cold response mechanism in IR64 indica rice variety through comparative transcriptome analysis
Source: BMC Genomics. 2020 Jun 24;21:425. doi: 10.1186/s12864-020-06841-2 (PMC7315535; doi:10.1186/s12864-020-06841-2)

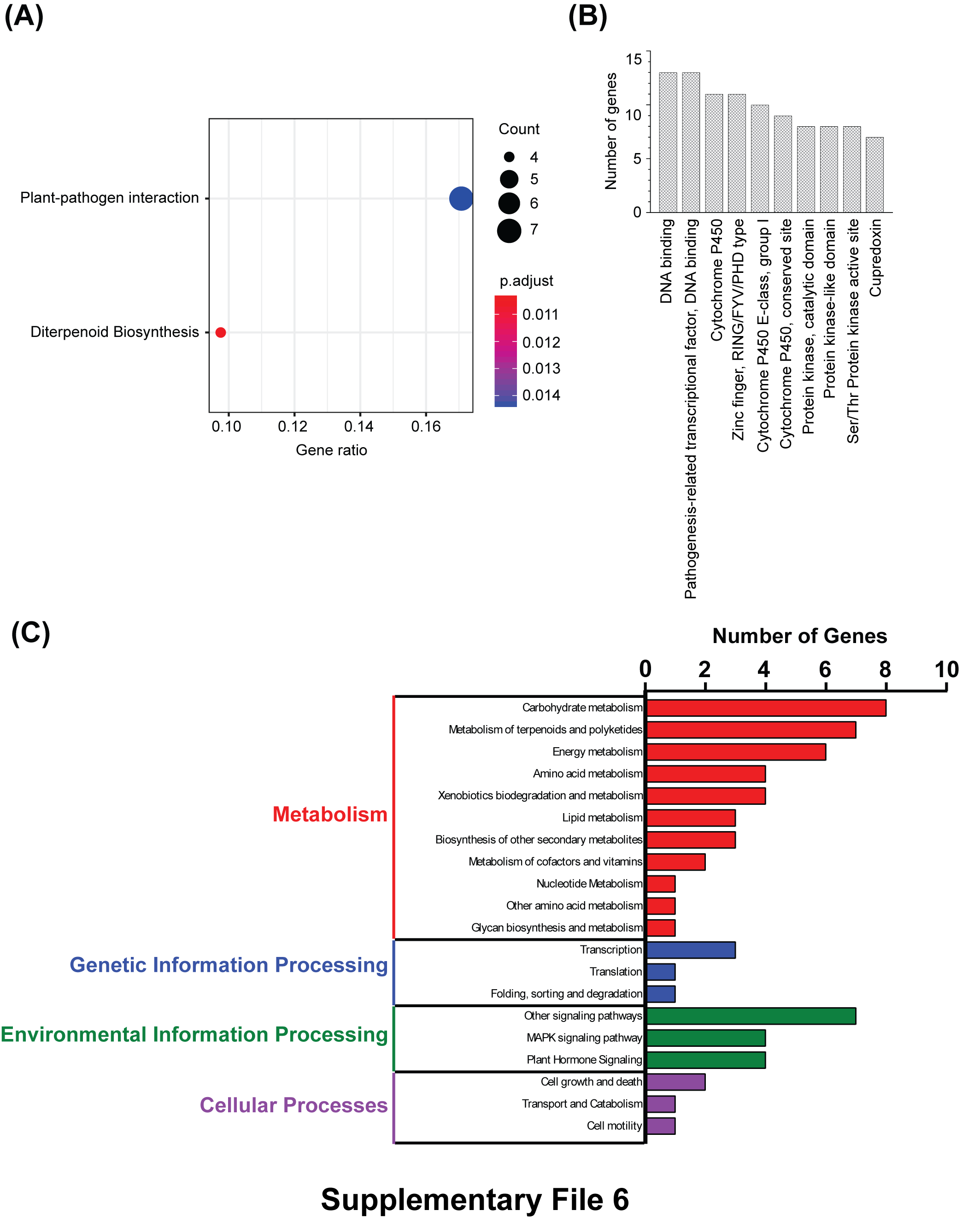

Supplement: Supplementary file 6 — Additional file 6. Functional annotation for differentially regulated genes. (A) KEGG pathway enrichment data for upregulated DEGs (B) Top ten abundant domains present in the upregulated genes, obtained using Blast2GO (C) the pathway reconstruction result of upregulated differentially expressed genes, based on KEGG, generated using BLASTKOALA. [file 12864_2020_6841_MOESM6_ESM.tif]
